# New Dielectric Sensors and Sensing Techniques for Soil and Snow Moisture Measurements

**DOI:** 10.3390/s90402951

**Published:** 2009-04-22

**Authors:** Markus Stacheder, Franz Koeniger, Rainer Schuhmann

**Affiliations:** Karlsruhe Institute of Technology (KIT), Competence Centre for Material Moisture (CMM), c/o Forschungszentrum Karlsruhe, Institut für Funktionelle Grenzflächen, Postfach 3640, 76021 Karlsruhe, Germany; E-Mails: franz.koeniger@ifg.fzk.de; rainer.schuhmann@ifg.fzk.de

**Keywords:** Electromagnetics (EM), Dielectrics, Time Domain Reflectometry (TDR), Frequency Domain (FD), soil moisture (SM), snow moisture

## Abstract

Measurements of material moisture are essential in fields such as agriculture or civil engineering. Electromagnetic techniques, more precisely dielectric methods, have gained wide acceptance in the last decades. Frequency or Time Domain methods take advantage of the high dielectric permittivity of water compared to dry materials. This paper presents four new dielectric sensors for the determination of soil or snow water content. After a short introduction into the principles, both the hardware and operating mode of each sensor are described. Field test results show the advantages and potentials such as automatic measurement and profiling, state-of-ground detection or large-scale determination. From the results it follows that the presented sensors offer promising new tools for modern environmental research.

## 1. Introduction

In nature and in many industrial processes, soil or material moisture is an important criterion and has great influence on natural and production processes. Although accurate determination of moisture is required by ISO standards, adequate and accurate techniques and methods are rare. The moisture determination of soil, many raw materials, foods, agricultural products and materials will help in many ways. Thus, soil moisture (SM) determination is an important issue when it comes to tillage, irrigation, applying fertilizers and harvesting. Moisture content of agricultural goods is essential concerning harvest, trading, transportation and storage. The water content is also a decisive criterion when it comes to natural hazards such as landslides, avalanches, mud streams and flooding events. Determination and survey of water content and soil saturation will help to reduce risks for people mainly in mountainous and riverine regions. Moisture controlled manufacturing processes will help to improve the quality and reduce losses during manufacturing and storage. This will help to save energy for example in drying, and will thus reduce pollution of the environment and improve quality of life.

Hence, adequate measurement systems are indispensable to properly assess the moisture of materials. Especially indirect methods based on electromagnetic (EM) principles have gained wide acceptance in the last decades for they can deliver fast, operational, *in-situ*, non-destructive and reliable measurements with acceptable precision.

The Competence Centre for Material Moisture (CMM) at the Karlsruhe Institute of Technology (KIT) is specialized on the development and testing of EM moisture sensors for various materials with a special focus on dielectric methods such as Time Domain Reflectometry (TDR) and Frequency Domain (FD) Methods. This paper gives a short review on the recent developments of new dielectric sensors and techniques for soil and snow moisture measurements of this institution. A short introduction to the basic measurement principles of dielectric measurement techniques will be given in the next paragraph.

## 2. Electromagnetic Moisture (EM) Measurement Methods

EM moisture measurement methods are so-called indirect methods that determine an electrical property which is closely related to the water content of the material, such as the electrical conductivity (EC) or the dielectric permittivity. Since EC is also considerably affected by the salinity of the material, mainly the dielectric methods promise good opportunities for accurate measurements.

There exists a broad variety of different dielectric moisture measurement methods [1]. Depending on the type of the material, the geometry of the sample, frequency range or desired accuracy, a suitable measurement method can be chosen. All methods are based on the interaction of an EM field and the material to be measured. A classification of the dielectric methods is possible by the way the EM field is generated and observed.

So-called FD methods on the one hand show a sinusoidal time dependence of the field. The steady state signals can be analysed by methods of the complex EM field theory.

TD methods, on the other hand, use signals with a transient character, confering a pulse-like time shape on the EM field. Both devices and analyzing techniques of these two methods differ significantly and are explained in more detail in the following.

### 2.1. Frequency Domain (FD) Technique

FD or capacitive techniques use capacitance to measure the dielectric permittivity of a surrounding medium and operate at one single measurement frequency. When the amount of water changes in the soil, a probe will measure a change in capacitance due to the change in dielectric permittivity that can be directly correlated with a change in soil water content [2]. During the last two decades, FD sensors have been gaining more and more acceptance in a broad range of agricultural, environmental, and engineering disciplines. Their widespread use has resulted partly from the simpler and thus less expensive electronics compared to TD methods. Currently, FD sensors are commercialized as single and multi-sensor capacitance probes with different installation and monitoring techniques [3].

### 2.2. Time Domain Reflectometry (TDR) Technique

TDR estimates the bulk dielectric permittivity, ε_b_, of the soil mixture (soil matrix, soil water and air) by measuring the propagation time of an EM pulse, generated by a pulse generator and containing a broad range of different measurement frequencies. The pulse propagates along a coaxial cable and enters the TDR probe, which is traditionally a pair of parallel metallic rods inserted into the soil. Part of the incident EM waves of the pulse is reflected at the top of the probe because of the difference in impedance between cable and probe. The remainder of the wave propagates through the probe until it reaches the end, where the wave is reflected back to its source. The transit time of the pulse for one round-trip, from the beginning to the end of the probe is measured with an oscilloscope branched on a cable tester. For a homogeneous soil, volumetric water content, θ_v_ (m^3^ m^−3^), is calculated by using a calibration curve which is normally established empirically with the desired material. One of the first and still widely accepted calibration functions for soils was established by Topp *et al*. at the beginning of the 1980s [4]:

(1) $$\theta_{v}=-5.3\times 10^{-2}+2.92\times 10^{-2}\varepsilon_{b}-5.5\times 10^{-4}\varepsilon_{b}^{2}+4.3\times 10^{-6}\varepsilon_{b}^{3}$$

In Equation (1):

θ_v_ is volumetric soil water content [m^3^ m^−3^]
ε_b_ is bulk soil dielectric permittivity [-].

## 3. Sensor Developments and Applications of CMM

### 3.1. FD Sensor ‘LUMBRICUS’

**Figure 1.**
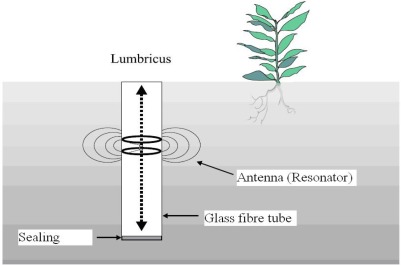
‘LUMBRICUS’ FD sensor with antenna, access tube and sealing.

One of the first developments of the former Soil Moisture Group (SMG), from which today’s CMM emerged, was a FD type sensor technique which is used by Meteoloabor AG in the *LUMBRICUS* SM device [5]. The portable moisture measurement system consists of a glass fibre access tube which will be inserted into the soil prior to the measurement and in which an antenna (resonator) can be moved up and down (Figure 1).

**Figure 2.**
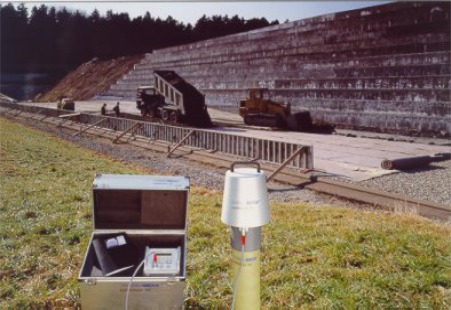
Commercial design of FD sensor ‘*LUMBRICUS*’.

The field of the antenna penetrates the tube walls into the soil and is influenced by its dielectric properties which lead to a shift in resonance frequency as well as a change of the amplitude and bandwidth. A voltage controlled oscillator controlled by a monoboard computer sweeps a frequency range of 100 MHz to 300 MHz. Attenuators improve the adjustment and reduce the noise of the signal and a diode detector measures the performance. The resonator-type antenna is coupled with this transmission path and in the case of resonance it acts as an absorption circuit and detracts energy. The remaining power and the corresponding frequency are measured and recorded by the computer. The resonator has a resonance frequency of 230 MHz for air and 170 MHz for saturated soil. Since the resonance curves are not only influenced by the real part of the dielectric permittivity but also by the quality, it is possible to determine the complex dielectric properties [6]. Figure 2 shows the commercial design of *LUMBRICUS*. On the right hand side the probe head with the integrated antenna on top of an installed access tube can be seen. The antenna can be moved automatically up and down and allows a water content profiling of the soil with a vertical resolution of 3 cm. The control module in the aluminium case operates the antenna movement and recording of the data.

### 3.2. TDR-Sensor ‘TAUPE’

**Figure 3.**
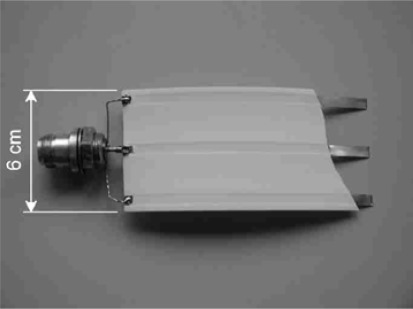
The PE-insulated flat band cable of the *TAUPE-TDR* sensor. (short section with uncovered conductor to show connection and geometry)

Another development of the CMM is a new moisture sensor for use with conventional TDR devices. But instead of conventional small scale TDR sensors with their rigid constructions and very limited, rather point measurement volume [7], a flexible polyethylene (PE) flat band cable with up to several m in length has been proposed (*TAUPE*), which can be used for large-scale, area-wide moisture measurements. Three copper stripes which act as the electrical transmission lines are inserted in the band. A picture of the cable is shown in Figure 3.

**Figure 4.**
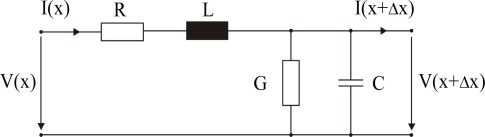
Electric equivalent circuit of an infinitesimal section of a TEM transmission line.

The electrical field concentrates around the conductors and defines the sensitive area of 3 to 5 cm around the cable. The spatial weighting of the measurements in the cross section of the cable is directly related to the energy density distribution. The electric properties of the cable can be calculated and measured. One can assume that the well-known equivalent circuit for the infinitesimal line section as shown in Figure 4 fully describes the electric properties of the line.

Beside SM measurements, this sensor is particularly applicable in dielectrically lossless materials such as snow. Better prognoses in avalanche and flood warning as well as in predicting the filling stages of Nordic hydro-power reservoirs require improvements in the determination of snow pack properties such as liquid water content, snow density and snow water equivalent (SWE). For this purpose the unshielded three-wire flat band cable sensors were connected to conventional TDR cable testers and a low frequency impedance analyser. The EM characteristics of the cable depend mainly on the dielectric properties of the snow pack and thus allowed their determination simultaneously, non-destructively and long-term.

The cables can have lengths of several 10 m, thus the covered area can be as large as a radar pixel size and a calibration of remote sensing for SWE determination is possible [8]. A new mode compensates for the effect of air gaps by measuring the three wire sensor cable with different penetration depths of the EM field, so that the gap has different influences on the measurement of the dielectrics and can be corrected [9].

With high frequency measurements and a suitable reconstruction algorithm [10], it was also possible to do moisture profiling along the cables to get information about spatial resolution of e. g. snow inhomogeneities or percolation zones.

### 3.3. State-of-Ground (SOG) Sensor

The SOG is a description of the condition of the soil surface such as for example dry, wet, frozen, or snow covered. The Deutscher Wetterdienst (German Weather Service, DWD) has defined and coded several characteristic SOG (Table 1) which are recorded daily at every meteorological station by visual observations. In order to simplify the monitoring and recording, there was the desire to develop a system that automatically detects these states of ground.

Since the majority of the different SOG is mainly influenced by the moisture and therefore show characteristic dielectric properties, we suggested to use the FD techniques to design a measurement sensor and device that is capable to determine the different states by a capacitance measurement.

**Figure 5.**
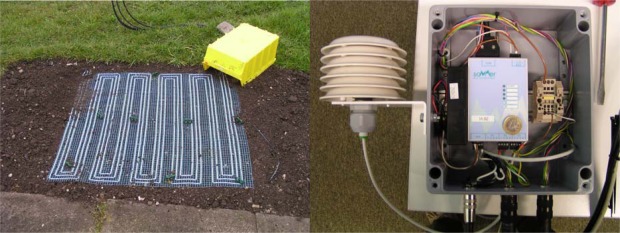
(left) Sensor mat for SOG detection. (right) IA in weather-proof box with an attached dew-point sensor.

The system consists of a 1 m^2^ large permeable sensor mat with interlaced electrode cables and an impedance analyser (IA), as shown in Figure 5.

At low frequencies (kHz-range), complex impedance or the variation of the capacitance of a capacitor can be measured by the IA using simple bridge and voltage divider switches. Based on circuit theory considerations and calculations [6], a field-compatible IA was designed. The core component is an embedded web module with a 16-bit 20 MHz 80186 CPU. Concerning the mode of operation, the measurement frequency is adjustable by software command and generated by the direct digital synthesis method. Sixteen different frequencies can be selected at a time and are measured stepwise in one measurement cycle. Measurement frequency is transformed to a measurement bridge connected to the sensor module. In the control unit, the magnitude and phase of the resulting voltage in the bridge are evaluated, digitised, calculated, and displayed via an interface. The IA is additionally equipped with 4 analogue outputs for the connection of temperature sensors or other electronic sensors.

### 3.4. Free-Line-Sensing (FLS) Technique

Large area integrated SM measurement results are often required for meteorological studies but also for geological, agricultural or natural disaster research. Yet conventional SM sensors measure mostly at localized points and thus reflect strong spatial variability due to the heterogeneity of most land surfaces.

**Figure 6.**
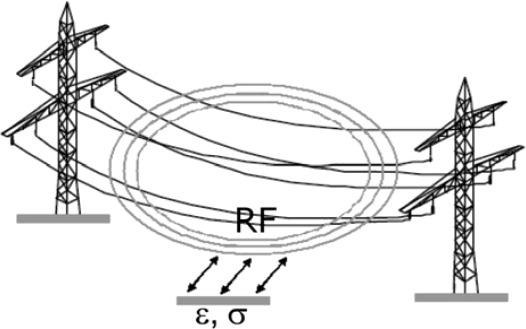
Schematic principle of the ‘FLS’ technique.

To overcome these disadvantages, the idea of the FLS technology is to use existing high voltage power lines of several km in length to detect variations in SM below the lines. The new method is based on the principle that every freely suspended metallic line acts as an antenna for guiding and radiating EM waves (Figure 6) and thus can principally be used as a probe for non-destructive area-wide SM sensing [11].

A high-voltage transmission line transports energy as alternating current (AC) at frequencies of 50 or 60 Hz, depending on standards defined by each country. The AC voltage is superimposed by small signals at frequencies of 50 kHz up to 500 kHz which can be evaluated for the moisture information of the soil below. When increasing SM leads to an increase in bulk soil dielectric permittivity, ε_b_, and bulk soil EC, σ_b_, this will affect the speed and the phase relation of the EM waves and thus influences the signals on the power lines. Alterations of amplitude and phase of the excited signals need to be recorded with a suitable measuring system.

**Figure 7.**
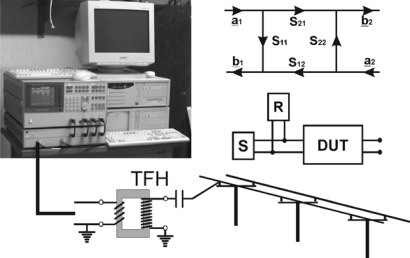
Measuring equipment for the ‘Free Line Sensor’ with a HP3588B vector network analyser, HP35677A S-parameter test set and personal computer. The meaning of the S-parameters is shown on the right; the measuring parameter is the input reflection factor S_11_ = b_1_ / a_1_.

The measurement equipment (Figure 7) consists of a highly stable signal source (S) which feeds the radio-frequency (RF)-signals between 50 kHz and 500 kHz into the power line and a sensitive receiver (R) which evaluates the magnitude and phase of the signals reflected by the power line (DUT, device under test). For this purpose, a HP3588B vector network analyser with a HP35677A S-Parameter test set was used. The amplitude of the continuous signal of the source is 1 V. A coupling circuit provides for the matching of the impedances between source and power line and the insulation from high voltage.

S-parameters describe the signal flow between the input and output of a DUT as a matrix [12]. The parameter S_11_, for example, describes the amplitude and phase change of an RF-signal on the power line as the ratio of a complex output variable b_1_ and the complex input variable a_1_ on port 1, while the parameter suffix 2 describes the signals on port 2. The definition of S-parameters is:

(2) $$S_{11}={\underline{b}}_{1}/{\underline{a}}_{1},S_{21}={\underline{b}}_{2}/{\underline{a}}_{1},S_{12}={\underline{b}}_{1}/{\underline{a}}_{2}\;\text{and}\;S_{22}={\underline{b}}_{2}/{\underline{a}}_{2}$$

To analyse very small amplitude and phase changes, it is essential to associate the input signal reflected by the power line with the output signal of the source. This synchronisation is difficult to achieve over long distances. Consequently, the input reflection factor S_11_ has been chosen for the FLS technique instead of the power transmission factor S_21_. The high dynamic range of the measuring equipment with a narrow bandwidth of 100 Hz provides for a good signal-to-noise ratio. The power line is matched with its characteristic impedance.

**Figure 8.**
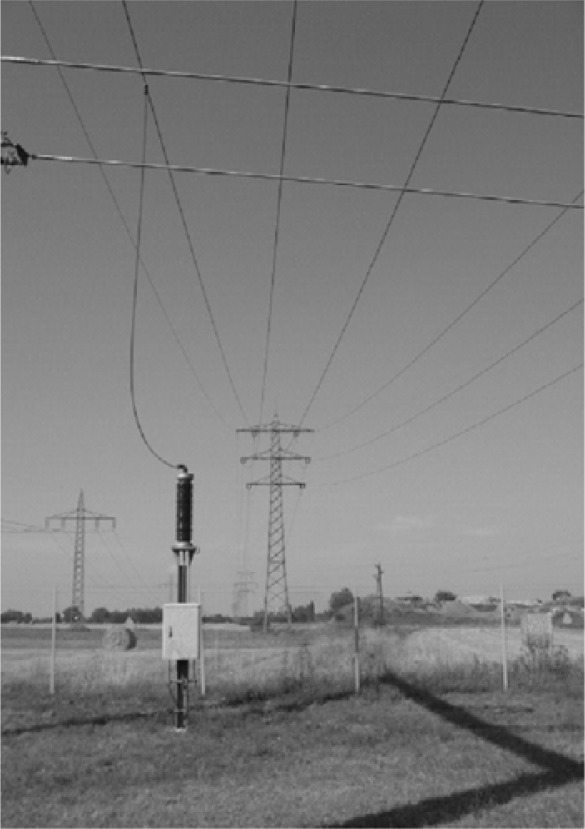
Signal coupling of the low-voltage signal to the high-voltage power line using a capacity post of the TFH equipment.

To protect the instruments from electrical damage, they have to be separated from high voltage (Figure 8). This was achieved with TFH equipment. TFH is a carrier frequency technique for telephony over high-voltage power lines [12] that has been used for decades by utility companies for control purposes and internal telephony and is also suitable for application in FLS.

## 4. Results and Discussion

### 4.1. SM Measurements with LUMBRICUS

**Figure 9.**
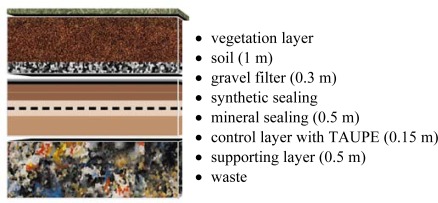
Schematic sketch of a surface barrier system of a waste disposal site.

The LUMBRICUS sensor was tested in a surface barrier system of a waste disposal site. The barrier system, shown in detail in Figure 9, consists of different mineral and artificial layers. Several access tubes of the LUMBRICUS system were installed in the barrier to monitor the change in water content within the different layers and several vertical moisture profiles were recorded.

**Figure 10.**
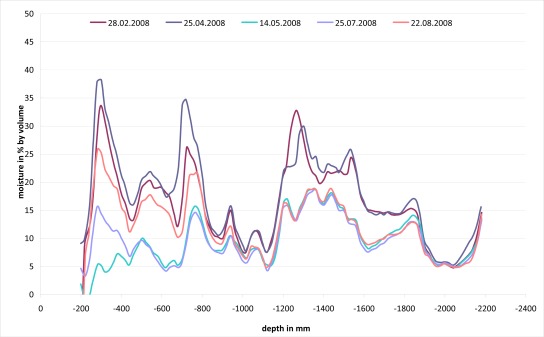
SM depth profiles of a surface barrier of a waste disposal site recorded with LUMBRICUS.

Figure 10 shows some measured moisture profiles of a single access tube at different days. The results are plausible and the different layers with their specific water contents are clearly distinguishable and the recorded water balance characteristics such as high variations in the upper soil layer due to precipitation events and low water content variations in the mineral sealing or the supporting layer are according to our experiences. The timely variations are also comprehensible and the correlation with reference measurements of soil samples by the oven-drying method was good.

### 4.2. TAUPE Snow Moisture Measurements

**Figure 11.**
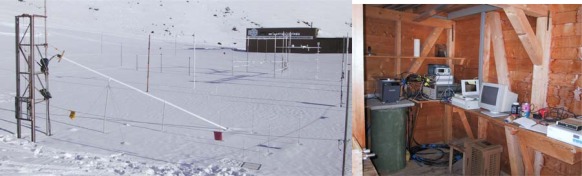
(left) High-elevation test site of ‘TAUPE-sensor’ at Weißfluhjoch, Davos, Switzerland. (right) Shelter with measurement devices.

The TAUPE sensor system was also tested for water content determination in a snow cover. The sensors were installed at a high-elevation field site in Switzerland (Figure 11) and at sites in Canada with different set-ups prior to winter and were enclosed by snowfall. The cables were mounted in a way to follow the natural settlement of the snow pack. The electronic measurement devices of the system were placed in the shelter approximately 10 m away from the cable sensors. For the high frequency measurements (100 MHz to 1 GHz) a ‘Tektronix 1502B’ TDR cable tester was used, while the low frequency measurements in the range of 1 kHz to 300 kHz were carried out with a ‘HP-VNWA 8712’ impedance analyzer. A PC and a self-made multiplexer completed the system. The measurements started on December 21, 2001 and ran automatically until the end of the winter season on June 21, 2002. Dielectric constant, snow density, and liquid water content of the snow packs were calculated from the raw signals. Also the moisture distribution along one of the horizontally laid out cables was reconstructed for different stages of the winter.

**Figure 12.**
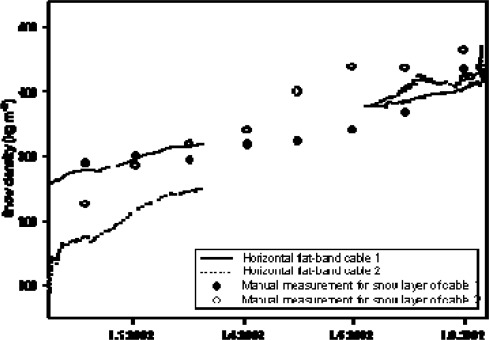
Measurements of snow density as a function of time. Comparison of TAUPE measurements at two different cables with manual measurements with snow cylinder.

The natural settling of the snow cover was reflected nicely in the horizontal cable measurements (Figure 12). The measured increase of snow density was in accordance with manual measurements. It increased from initially 100–200 kg m^−3^ to approximately 500 kg m^−3^ at the end of the winter season.

The two cables located at two different depths of the snowpack also reproduced correctly a higher density in the lower part of the profile in February (cable 1) and a faster compaction in the upper part during the snowmelt starting in early May (cable 2). However cable 2 seems to have systematically underestimated density, which we think may be explained by spatial differences in the measurement spots of cable and manual density measurements. The determined liquid water content (LWC) in the melting season gave plausible results too, both compared to lysimeter data taken on the test field and with regard to the spatial variation of flow fingers that are normally experienced in a natural snow pack [14].

**Figure 13.**
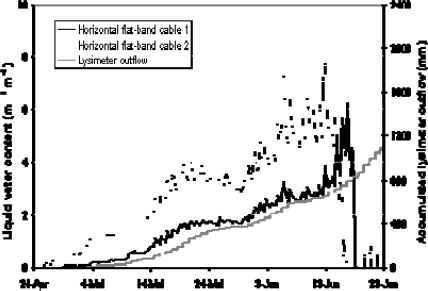
Measurements of LWC in a snowpack as a function of time. Comparison of two TAUPE cables with lysimeter data.

No LWC was detected with the horizontal cables until the end of April, when the snowpack had reached its maximum depth (Figure 13). This was supported by automated snow temperature measurements, which indicated dry snow conditions and temperatures well below zero before this period. Once the snowmelt set in, we measured the steadily increasing LWC with the horizontal cables indicating the downwards penetration of the wetting-front. The upper cable (no. 2) not only reflected an earlier and faster increase in liquid water content than the lower cable, it also reacted more clearly to the diurnal variation caused by melting during the day and refreezing during the night. The calculation of the snow water equivalent from this data, i. e. the product of snow density and snow depth or, in other words, the amount of water the snow pack would give when melted, gave encouraging results.

**Figure 14.**
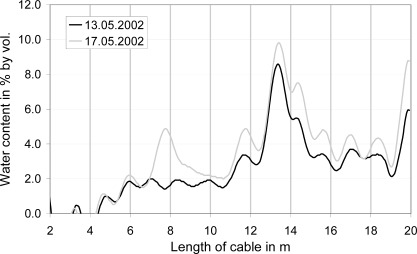
Spatial variation of LWC along the upper horizontal cable at two days during the melting period.

With regard to the spatial variation of LWC, the horizontal flat-band cables nicely demonstrated the formation of preferential water flow paths adjacent to the cable as shown in Figure 14 for one cable at two different days in the melting season. The horizontal cable at about 1 m below the snow surface indicated in mid May an emerging water conducting zone at 14 m from the cable end, as well as a newly developing flow finger at 8 m from the cable end. The formation of flow paths in the snow is a well known natural process of water transport in a melting snowpack [14] and these results clearly demonstrate the excellent capabilities for the spatial resolution of the measurements by the suitable reconstruction algorithm established by [10].

### 4.3. Measurements with SOG Sensor

**Figure 15.**
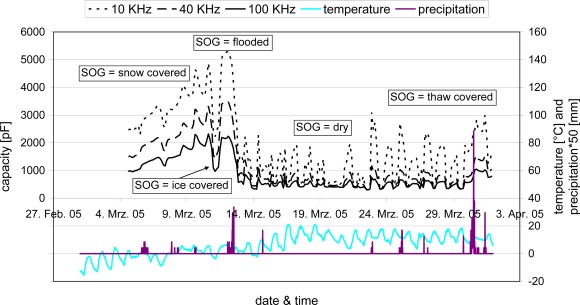
Time-dependent capacities at selected frequencies as well as reference values of temperature and precipitation for different SOG in the month of March 2005.

The SOG sensor and device were tested in the field on a natural undisturbed soil surface from which only the vegetation layer was removed. As a reference for the assessment of the measurement results nearby measurements of air temperature, precipitation and visual observations from a meteorological station were used. Figure 15 shows the measured capacity of the impedance analyser at three different frequencies (10, 40, and 100 kHz) as a function of time for the month of March 2005.

The month started with a considerable snow cover, a ‘flooded’ (E3) SOG was reached after snow melt and heavy rainfalls. It led to capacities higher than 2,000 pF at 100 kHz frequency. A short cold snap in the night of March 11 froze the flooded parts and resulted in an ‘ice-covered’ (E5) SOG. After thawing and again flooding, the soil gradually dried and the large differences in temperature between day and night regularly led to thaw covers (EE3), clearly visible by little peaks of the capacities at the start of the day.

It gets obvious that each of the described SOG shows characteristic capacitances and with the help of the reference measurements a decision matrix with several criteria, such as the measured soil capacitance at three different frequency, the temperature, and SOG history, could be established [15]. It allows for the interpretation of the measurements and, hence, an automatic determination of the SOG. The new system proved to be reliable and field-capable so that it could be a promising new tool for the automatic characterisation of the SOG, for example for weather forecast and traffic control services as well as for agricultural applications.

### 4.4. Large-Scale-SM Measurements with FLS Technique

A preliminary field test of the FLS technique was conducted on a 220 kV power line in the Rhine valley in the South of Karlsruhe, Germany. The power line was approximately 13 km long extending over different terrain types with soil types ranging from silty sand to clayey loam and partly swampy terrain with near-surface groundwater. The average distance of the power line to the ground was at approximately 12 m.

**Figure 16.**
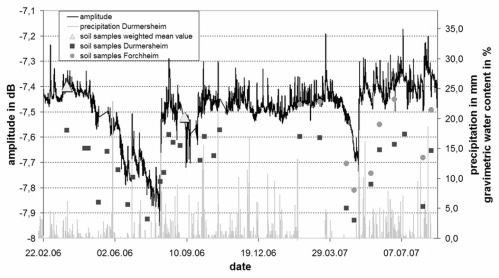
Amplitude of the reflected signal and SM at two sites below the power line. The weighted mean values (triangles) of SM taken along the complete transect are added.

Test measurements were performed at different suitable frequencies and the results were compared to precipitation measurements from rain gauges and moisture data from soil samples. The signals for all frequencies were in good agreement in terms of both amplitude and phase. A representative result of the reflected signal at 165 kHz is presented in Figure 16 as a time series of amplitude measurements with precipitation events recorded at the location of the test site. The amplitude of the RF-signal data was scaled to the weighted mean values.

**Figure 17.**
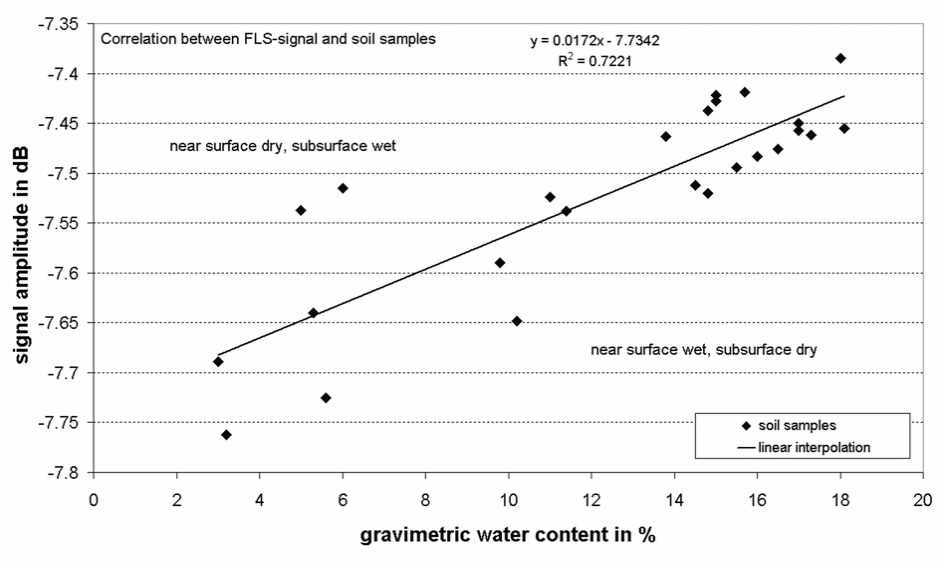
Correlation between the GWC of soil samples and the FLS signal.

During precipitation events, when the soil at the surface is nearly saturated, the amplitude of the detected signal rises significantly followed by a slow decrease when the subsurface dries again. The data reveal a good correlation between rapid amplitude changes and precipitation events. Between the end of April and the end of July 2006 there is a strong decrease of the curve reflecting long periods of negligible precipitation and high evapotranspiration. This period is followed by a stable situation of relatively wet soil with high amplitude values until the end of the year 2006 and beginning of 2007. Remarkable is also the sharp decrease in April 2007 with no precipitation recorded and entering the annals as warmest April ever in this region. Figure 17 shows the relationship between the amplitude of the Free Line Sensor measurements at a frequency of 165 kHz and the gravimetric water contents (GWC) of the soil samples.

The weighted mean values of GWC were scaled to the measured values of the RF-signals as follows:

(3) GWC [%]=44.346 [%/dB]*signal amplitude [dB]+353.658 [%]

The graph reveals one of the main technical difficulties of the method: as the penetration depth of the RF-signals (> 1 m) exceeds the sample depth range (0.1 −0.3 m) it varies with the influence of the dielectric properties, hysteresis is found in the signal. If the near-surface SM is low, but water content in the subsurface is high, the FLS overestimates the water content. If, on the contrary, the moisture content of the subsurface, e. g. in summer, is low, and heavy rainfall occurs, the electrical signal is too low to give water contents comparable to the reference. This explains why the correlation factor R^2^ is only 0.72.

The integrated measurement of FLS results in a weighted mean of the SM over an area of several kilometers below the power line, if both the variation of the environment and the strength of the EM fields are taken into account. Due to the small-scale variability of the terrain and strong local differences in SM, further investigations will have to be carried out to adjust the results of the measurements with the FLS to the actual integrated moisture content. Also additional effects resulting from the variations of the line distance from the ground due to sloped terrain, seasonal vegetation, or line variations due to temperature have to be taken into account. But nevertheless the presented results demonstrate the capability of this technique for soil surface monitoring.

## 5. Conclusions

Some important EM sensor developments and new sensing techniques of the Competence Center for Material Moisture (CMM) of the Karlsruhe Institute of Technology (KIT) for the characterization of the soil, the soil surface and its cover have been presented. The particular measurement methods as well as the designed measurement hardware have been introduced. All developments are based on the determination of the dielectric properties of the material, which are mainly influenced by the moisture due to the high dielectric permittivity of water.

Two sensor techniques, the *LUMBRICUS* Moisture Meter and the SOG sensor, are based on the FD technique which determines the capacity as a function of the dielectric permittivity. The *TAUPE* sensor uses the TDR technique to derive the moisture of a material and the FLS technique measures amplitude and phase changes of EM waves on electrical transmission lines by the surrounding material.

The presented sensors and techniques were thoroughly tested in the field. The measurements were evaluated and some interesting results were presented such as the SM profiling method via access tubes with *LUMBRICUS,* the determination of important snow parameters such as density or liquid water content with *TAUPE* sensors, the characterization of the soil surface with the SOG sensor or large-scale SM interpretation with the FLS technique. Most measurement results were in close agreement with suitable reference measurements or well-established experiences and revealed the potential of these new techniques and sensors for modern environmental research such as operational, non-destructive and automatic measurements with good monitoring and networking capabilities as well as problem-specific measurement solutions such as in-situ determination and area-wide characterization of important soil parameters.

## Figures and Tables

**Table 1.** Different SOG with corresponding codes of the DWD and its descriptions for visual observations.

**State of ground without deposits**	**DWD code**	**Description**
dry	E 0	state of soil after long period without precipitation, no bonding of soil particles visible, presence of dust
moist	E 1	grains of sandy soils bonded together, silty soils show larger formable aggregates
wet	E 2	water puddles accumulate on the ground, soil can be impressed easily, dents fill with water
flooded	E 3	large parts of the soil are covered with water, which cannot drain away
frozen	E 4	former wet soils at temperatures below 0 °C, soil hard and cannot be impressed
ice-covered	E 5	liquid precipitation falls onto cold surface or under-cooled precipitation falls onto ground and freezes, also melt water and poodles can lead to ice cover during cooling
dust-covered	E 6	loose cover with dust or sand
very dry	E 9	shrinking cracks observable mostly on silty or clayey soils
**State of ground with deposits**		

graupel or hail	EE 0	more than 50% of surface covered with hail, graupel, ice or snow grains
snow	EE 1	more than 50% of surface covered by snow
hoar	EE 2	discharged precipitations of hoar onto ground
thaw	EE 3	discharged precipitations of thaw onto ground
